# The Prevalence and Impact of Fake News on COVID-19 Vaccination in Taiwan: Retrospective Study of Digital Media

**DOI:** 10.2196/36830

**Published:** 2022-04-26

**Authors:** Yen-Pin Chen, Yi-Ying Chen, Kai-Chou Yang, Feipei Lai, Chien-Hua Huang, Yun-Nung Chen, Yi-Chin Tu

**Affiliations:** 1 Graduate Institute of Biomedical Electronics and Bioinformatics National Taiwan University Taipei Taiwan; 2 Department of Emergency Medicine National Taiwan University Hospital Taipei Taiwan; 3 Taiwan AI Labs Taipei Taiwan; 4 Department of Computer Science and Information Engineering National Taiwan University Taipei Taiwan

**Keywords:** misinformation, vaccine hesitancy, vaccination, infodemic, infodemiology, COVID-19, public immunity, social media, fake news

## Abstract

**Background:**

Vaccination is an important intervention to prevent the incidence and spread of serious diseases. Many factors including information obtained from the internet influence individuals’ decisions to vaccinate. Misinformation is a critical issue and can be hard to detect, although it can change people's minds, opinions, and decisions. The impact of misinformation on public health and vaccination hesitancy is well documented, but little research has been conducted on the relationship between the size of the population reached by misinformation and the vaccination decisions made by that population. A number of fact-checking services are available on the web, including the Islander news analysis system, a free web service that provides individuals with real-time judgment on web news. In this study, we used such services to estimate the amount of fake news available and used Google Trends levels to model the spread of fake news. We quantified this relationship using official public data on COVID-19 vaccination in Taiwan.

**Objective:**

In this study, we aimed to quantify the impact of the magnitude of the propagation of fake news on vaccination decisions.

**Methods:**

We collected public data about COVID-19 infections and vaccination from Taiwan's official website and estimated the popularity of searches using Google Trends. We indirectly collected news from 26 digital media sources, using the news database of the Islander system. This system crawls the internet in real time, analyzes the news, and stores it. The incitement and suspicion scores of the Islander system were used to objectively judge news, and a fake news percentage variable was produced. We used multivariable linear regression, chi-square tests, and the Johnson-Neyman procedure to analyze this relationship, using weekly data.

**Results:**

A total of 791,183 news items were obtained over 43 weeks in 2021. There was a significant increase in the proportion of fake news in 11 of the 26 media sources during the public vaccination stage. The regression model revealed a positive adjusted coefficient (β=0.98, *P*=.002) of vaccine availability on the following week's vaccination doses, and a negative adjusted coefficient (β=–3.21, *P*=.04) of the interaction term on the fake news percentage with the Google Trends level. The Johnson-Neiman plot of the adjusted effect for the interaction term showed that the Google Trends level had a significant negative adjustment effect on vaccination doses for the following week when the proportion of fake news exceeded 39.3%.

**Conclusions:**

There was a significant relationship between the amount of fake news to which the population was exposed and the number of vaccination doses administered. Reducing the amount of fake news and increasing public immunity to misinformation will be critical to maintain public health in the internet age.

## Introduction

To take the blue pill or the red pill: decisions are made every day in our lives. As expressed in the 1999 film The Matrix, “You take the blue pill—the story ends, you wake up in your bed and believe whatever you want to believe. You take the red pill—you stay in Wonderland, and I show you how deep the rabbit hole goes” [1]. Every decision may have critical or trivial effects on our future and may be influenced by our environment. Decisions about whether to accept or reject vaccination can be influenced by a variety of factors [2-6] including personal lifestyle, disease severity, vaccine effectiveness, side effects, peer decisions, and internet information. The internet has brought everyone together over the last decades, and misinformation on the internet can spread like a plague and affect public positions [7-13], even encouraging individuals to make potentially self-harming health decisions [14,15].

**Figure 1:**
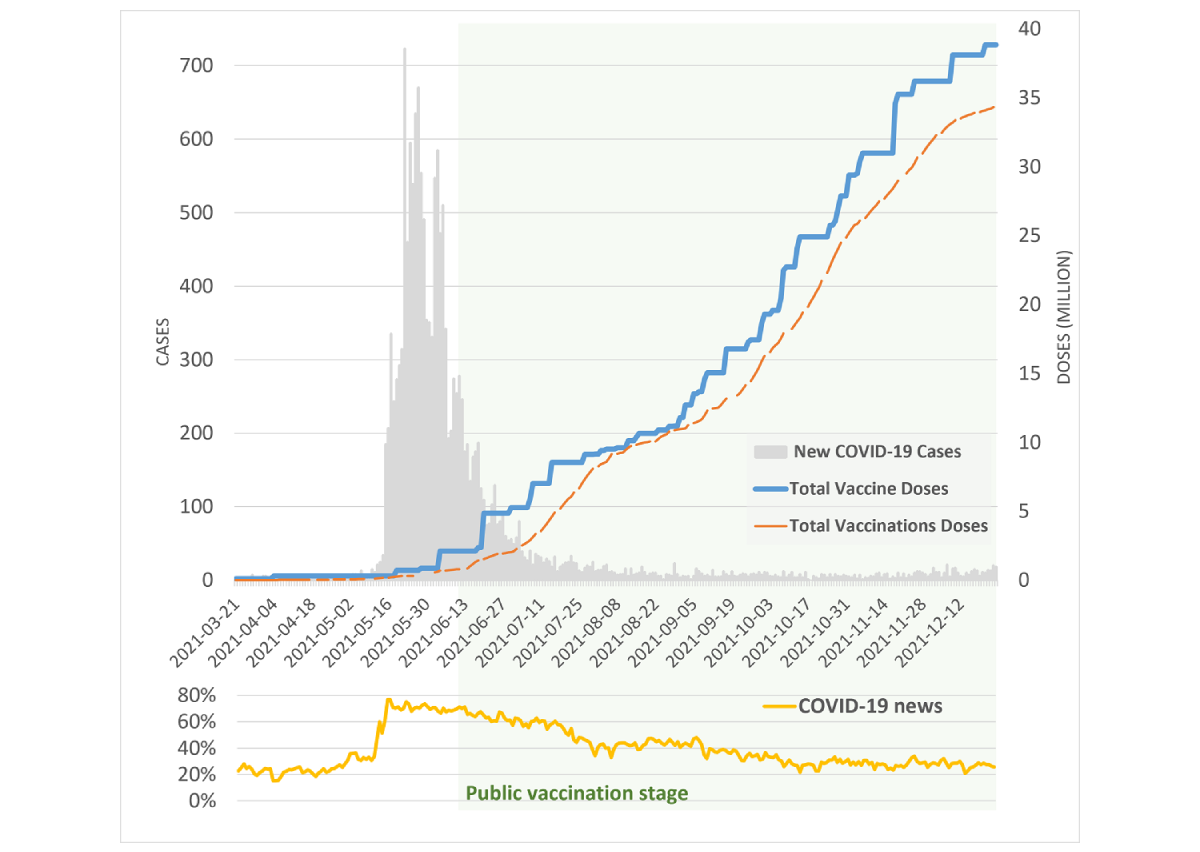
Data about COVID-19 infection cases, total vaccine doses, vaccine uptake (vaccination doses), and the percentage of COVID-19 news in Taiwan. The data covers a period ranging from March 2021 to December 2021, and the orange dotted line represents vaccinations in Taiwan, with missing values on weekends and holidays. The public vaccination stage began on June 12, 2021, as indicated by the green background.

The COVID-19 pandemic spread around the world from about mid-2020, and vaccines were authorized for emergency use in early 2021 [16]. Taiwan, located in East Asia, with a population of 23 million (population density of 646 people/km²), received its first batch of COVID-19 vaccines on March 3, 2021, and started vaccination on March 22, 2021 [17]. Given the initially limited number of vaccine doses available, and the policy to vaccinate health care workers first, public vaccination started on June 12, 2021 [17]. During the vaccination period, Taiwan experienced its first wave of large-scale community infections, and the internet was flooded with news about COVID-19 and vaccines (Figure 1). Considerable research has indicated that misinformation about diseases and potential vaccine side effects have adverse effects on vaccination rates [15,18,19]. Some researchers have designed questionnaire-based studies to investigate this association [20-22]. One such study quantified the rise in the number of antivaccine tweets during the pandemic [23], and several studies investigated factors affecting the spread of misinformation [24,25]. Building upon this previous research, we hypothesized that a higher prevalence of misinformation might have a greater adverse effect on vaccination decisions.

Detecting misinformation or fake news from big data on the internet is challenging [13]. In this decade, deep learning for natural language processing (NLP) has been developed to help address this problem, and many news analysis services are already available on the web [26]. These services use machine learning algorithms or manual detection methods to provide online fact-checking covering multiple topics [26,27]. However, these services were difficult to use in this study due to language differences. In this study, we focused on digital media news in Taiwan and used the Islander news analysis system [28], which uses an innovative language model to automatically screen and score internet news.

**Figure 2:**
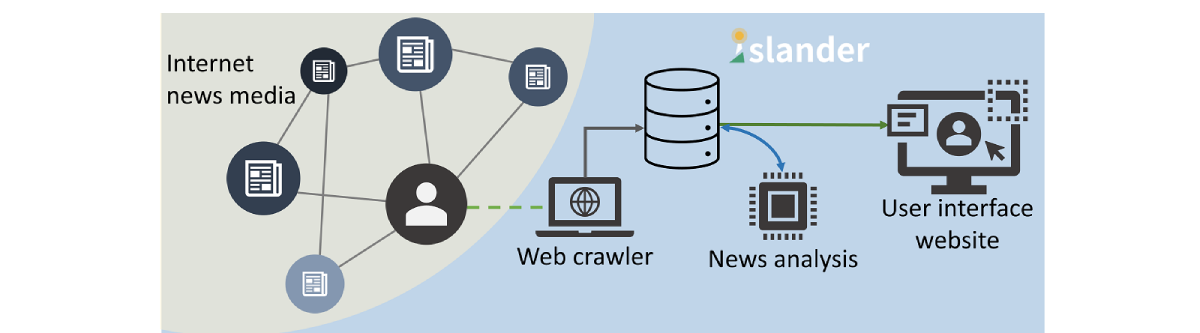
The Islander news analysis system. This system has 3 components: a web crawler to collect web news in real time, a news analysis model to judge the news objectively, and a website that provides a user interface.

There is no consistent definition of fake news; its identification is complex and can sometimes be difficult to determine [12,27,29-31]. The definition of fake news can be as broad as improper information or stories [18,27,32], or as narrow as verifiably false articles deliberately published by the media [11,12,27], and anything in between [13,33]. Experts or the wisdom of crowds can detect false information manually [27,34], but efficiency can be an issue when news may have spread before a judgment was made. An automatic detection method could involve knowledge base retrieval systems [27], but breakthrough knowledge may be considered misinformation. Content style analysis is another automated method, based on the assumption that there is a certain pattern in intentional news [31,35-37], but outlets may evade detection by manipulating their writing style [27]. In this study, we employed a style-based approach to fake news detection. Generally speaking, the typical characteristics of fake news are associated with the writing style, quantity of subjective language, and sentiment lexical or incited discourse [26,27,31,35-37]. We adopted the scores of suspicion and incitement provided by the Islander news analysis system [28] in which a language model, RoBERTa [38], was trained using a supervised learning approach to analyze and score news (Figure 2). This news analysis language model was trained on the Chinese valence-arousal text data set (CVAT) [39], and 198 random news items from mid-2019, labeled by 2 journalism experts. These 2 experts labeled the bias of the title and objective statements or subjective claims, and crossvalidated them. CVAT includes 720 texts tagged with affective words, and each sentence was scored according to valence and arousal, which were used to train the incitement judgment of the Islander system. This quantifiable domain knowledge, combined with the writing style and incited score, constitutes the Islander system's fake news discriminator.

Individuals obtain internet information by passively accepting pushes from web services or by actively searching for specific terms. Searches reflect user interests [40,41], and many web news services have adopted a recommendation system to push information to potentially interested people using data gathered from personal surfing behavior or search histories [13,42-44]. Some studies have indicated that search trends can reflect the amount of information dissemination [45,46]. We used Google Trends as a metric for the amount of information propagated by web news, due to its up to 85% market share [6,47].

**Figure 3:**
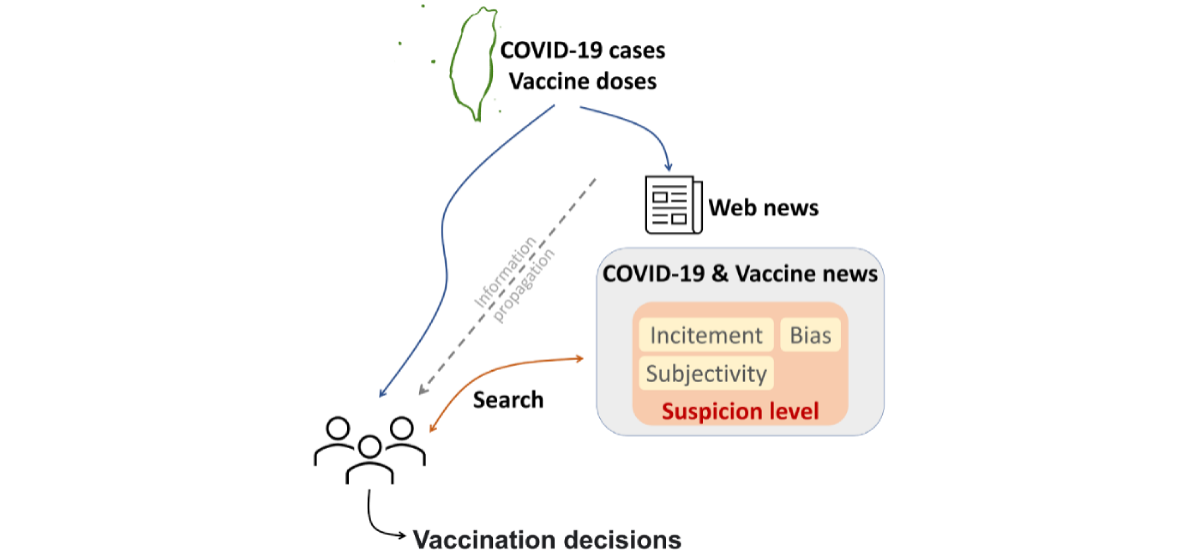
Graphical summary of this study. Taiwanese officials publicly release COVID-19 and vaccination information, and the media post news about this information on the internet. The public may obtain relevant information using searches or pushes from a recommendation service. This information will help individuals make vaccination decisions. In this study, we investigated the relationship between the quality of news, its dissemination, and vaccination decisions.

Few studies have investigated the interplay among the quantity of misinformation, information propagation, and its impact on decision-making [13]. In this study, we retrospectively analyzed the relationship between vaccination acceptance and digital news dissemination in Taiwan and aimed to quantify the effect of the propagation of fake news on COVID-19 vaccination decisions (Figure 3).

## Methods

### Study Design and Setting

The study population was the population of Taiwan. We conducted a retrospective study using publicly available data from March 1, 2021, to December 25, 2021, starting from when Taiwan first obtained the vaccine. The government publicly releases information about COVID-19, vaccines, and vaccination numbers, and we collected information on the COVID-19 pandemic from the Taiwan Ministry of Health and Welfare [17] and the Our World In Data [48] website. A total of 5 variables were used, including the number of COVID-19 infection cases, the number of COVID-19 deaths, total vaccine doses available, total vaccinations, and the number of vaccinated individuals. The web news we collected came from the Islander system news database in which news is crawled and stored in real time. Each news item included the title, content, source, publishing time, suspicion score, and incited score. We obtained data on daily trends through a Google Trends news subgroup search for “疫苗” (vaccine) in Taiwan within the date range.

**Figure 4:**
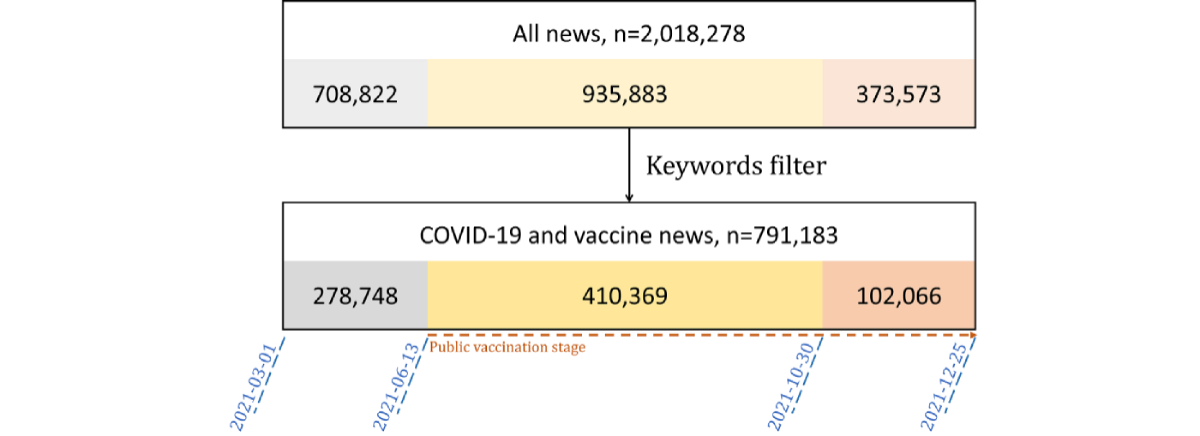
The news collected in this study. A total of 2,018,278 items were included and filtered by keywords for COVID-19 and vaccine news, leaving 791,183 news items for research. A study interval of June 13, 2021, to December 25, 2021, was used to investigate decisions by the public about vaccination. We used data from October 31, 2021, to December 25, 2021, for validation.

To investigate the relationship between internet news and vaccination acceptance by the public, we set the analysis interval from June 13, 2021, to December 25, 2021, according to the timing of public vaccination. We divided the time interval into training and validation parts, with a ratio of 70 to 30. Data from before October 30, 2021, were analyzed separately, and the other data were used for validation (Figure 4).

### Variables and Outcome

We resampled daily to weekly data and obtained the following information: the number of available vaccine doses, calculated as the difference between the number of vaccine doses available and the number of vaccinations; the number of new COVID-19 cases per week; the number of new COVID-19 deaths per week; the number of new vaccinations administered per week; the number of newly vaccinated people per week; and the average Google Trends score each week. Individuals will be interested in the issue and search for it, and relevant information will be provided; thus, we selected COVID-19 and vaccine keywords to filter the news data set. We filtered news related to COVID-19 and vaccination using the following keywords limited to Chinese news: “破口,” “病例,” “polymerase chain reaction (PCR),” “放寬,” “疫,” “隔離,” “確診,” “COVID,” “新冠,” “新型冠狀病毒,” “肺炎,” “疾管,” “疫苗,” “BioNTech (BNT),” “AstraZeneca (AZ),” “高端,” “默德納,” “Moderna,” “vaccine,” “接種,” “vaccinate,” “vaccination.” Multimedia Appendix 1 presents the meaning and English translation of the Chinese search keywords. Subgroups of digital news with different subsets of keywords were also employed in the study to investigate their relationship with vaccination doses. We counted the weekly number of news and the percentage of fake news. In this study, fake news was set as news with a suspicion score greater than zero. Suspicion scores ranged from 0 to 1000; lower scores indicate greater objectivity, and zero scoring was predominant in the data, which looked like a Poisson distribution. We also selected the weekly average incitement score as a variable. Incitement scores ranged from 0 to 1000 and presented as a Gaussian distribution; lower scores indicate less incitement (Multimedia Appendix 2).

The outcomes of this study were the number of new vaccination doses and newly vaccinated people for the following week. We investigated the factors affecting vaccination decisions using the following variables available: vaccine doses, new COVID-19 cases, average Google Trends score, fake news percentage, average incitement score, and the interaction term of the average Google Trends score with the fake news percentage.

### Statistical Analysis

We used chi-square tests for the analysis of fake news percentages, and multivariable linear regression with the stepwise method was used for variable selection. The variance inflation factor was used to detect multicollinearity among variables and to remove probable linear combinations of variables. The Johnson-Neyman procedure was used to generate plots of the interaction effects with 95% CIs. The final models were validated using the validation data.

Data were normalized and then analyzed using the R (version 4.1.1; R Core Team), statistical packages interactions (version 1.1.5), R commander (version 2.7-1), and RStudio (version 1.3.1093). All *P* values in this study were 2-sided and were considered statistically significant when less than .05.

## Results

**Figure 5:**
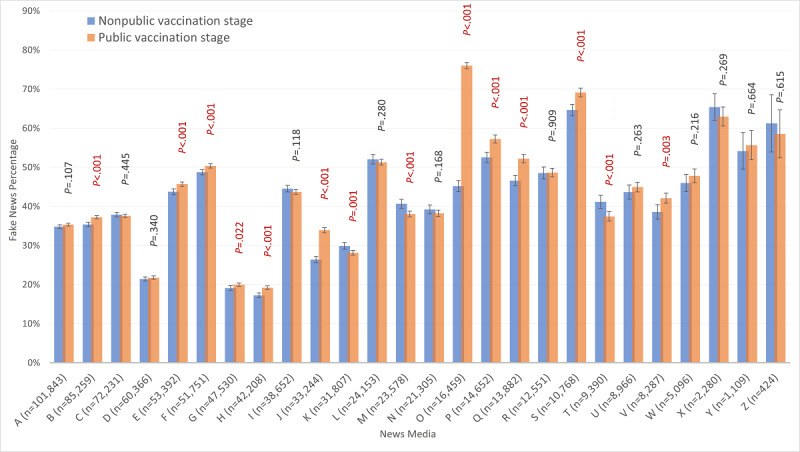
Fake news percentages, with 95% CI, of each media. Multimedia Appendix 1 provides the sources of digital media.

Using the settings described, 791,183 COVID-19 and vaccine news items were collected from 26 internet news media sources. A higher percentage of fake news (193,188/512,435, 37.7%; 95% CI 37.6%-37.8%) was found during the public vaccination stage, than during the nonpublic vaccination stage (99,791/278,748, 35.8%; 95% CI 35.6%-36.0%); and 11 of the 26 news media sources had significantly increased fake news percentages during the public vaccination stage (Figure 5). This study involved 28 weeks of data for the regression analysis (details on variables and outcomes are shown in Table 1). Every week, about 3 million vaccine doses were available in Taiwan, and about 1 million doses were administered to the public.

**Table 1:** Summary statistics of the variables used in the study.

		Mean (SD)	Minimum	Median	Maximum
**Variables**					
	Available vaccine doses	3,129,315.5 (1,684,054.2)	351,662	3,291,468	6,263,838
	New COVID-19 cases	148 (238)	28	65.5	1150
	New COVID-19 death cases	15.7 (30.4)	0	2	127
	Incitement score	488.7 (2.4)	483.9	488.5	492.4
	Fake news (%)	37.4 (1.9)	33.7	37.6	41.3
	Google Trends	22.4 (14.9)	4.3	19	54
**Outcomes**					
	Following week’s vaccination doses	1,194,379.4 (632,178.6)	308,400	1,090,186.5	2,764,054
	Following week’s newly vaccinated people	633,134.8 (490,868.2)	52,519	460,499.5	1,590,232

Multivariate analysis revealed a statistically significant relationship between the number of vaccine doses administered and the number of available vaccine doses, as well as an interaction term for the percentage of fake news and Google Trends levels. These significances persisted even when analyzed together with the validation data (Table 2). These coefficients suggested that there may be a positive relationship between the number of vaccine doses available and the number of vaccine doses administered during the following week, and that the incitement score might adversely affect vaccination doses in the following week. There also appeared to be an interaction between the fake news percentage and the Google Trends level, due to the opposite sign of the interaction term.

**Figure 6:**
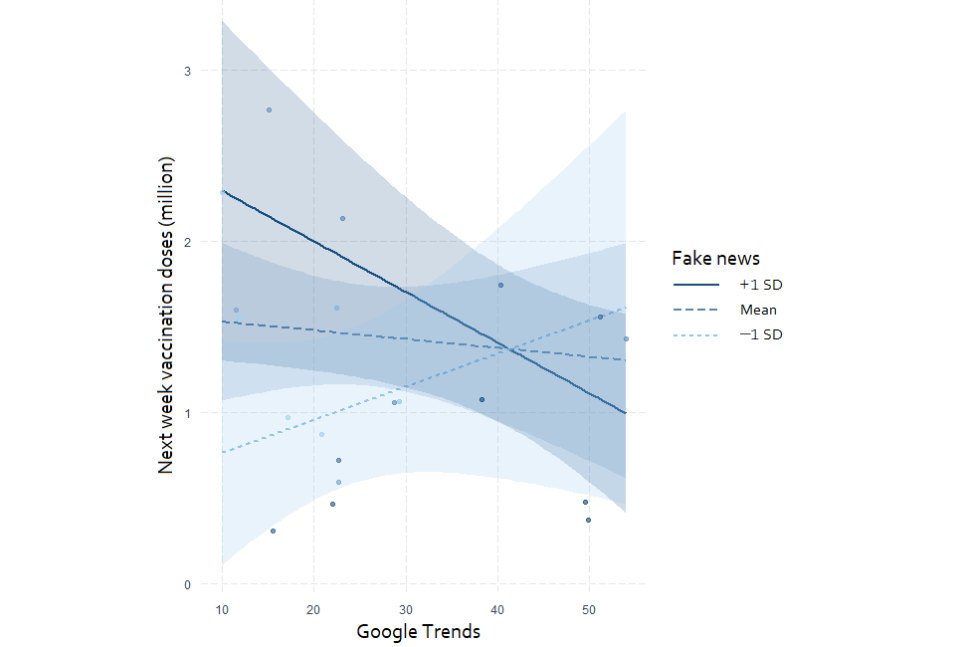
Interaction plot with 95% confidence bands. This plot demonstrates the interaction of the following week’s vaccination doses with the Google Trends levels for those with 1 SD above and below the average for the fake news percentage.

**Figure 7:**
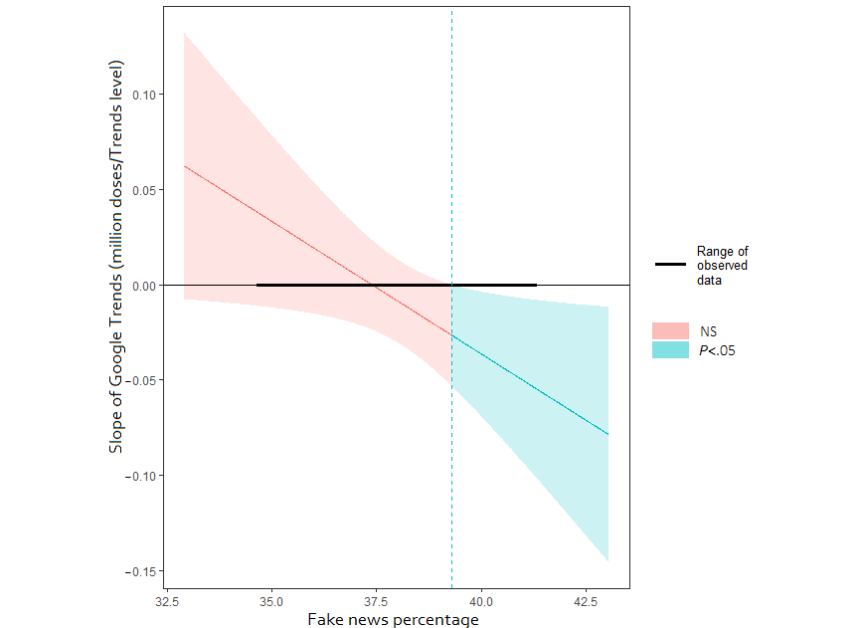
Johnson-Neyman plot with 95% confidence bands. This plot shows the Google Trends level coefficient adjusted for different percentages of fake news. NS: not significant.

The interaction effects for fake news percentage and Google Trends levels in the multiple regression revealed that as the fake news percentage increased, the slope of the Google Trends level moved from positive to negative (Figure 6). The Johnson-Neyman procedure suggested that when the fake news percentage exceeded 39.3%, the Google Trends level had a significantly negative adjusted effect on the following week's vaccination doses (Figure 7).

**Table 2:** A multivariable linear regression model of factors associated with vaccination doses for the following week. The variance inflation factor (VIF) for each factor was less than 10.

			June 13 to October 30, 2021^a^									June 13 to December 25, 2021^b^						
			Estimate		SE		*P* value		VIF		Estimate			SE		*P* value		VIF
**Coefficients**																		
	Intercept	–0.1482		0.4805		.76		—^c^		–0.0450			0.3721		.90		—	
	Available vaccine doses	0.9799		0.2637		.002^d^		1.96		0.4510			0.1774		.02^d^		1.43	
	Incitement score	–0.4725		0.2953		.13		3.31		–0.5222			0.2279		.03^d^		2.40	
	Fake news (%)	3.8286		1.9884		.07		4.72		1.6420			1.1771		.18		2.53	
	Google Trends	0.8257		0.5208		.14		8.14		1.0382			0.3970		.02^d^		6.64	
	Fake news: Google Trends	–3.2121		1.3796		.04^d^		9.95		–2.5846			0.9058		.009^d^		5.23	

^a^Multiple *R*^2^=0.647, adjusted *R*^2^=0.521, F_5,14_=5.133; *P*=.007.

^b^Multiple *R*^2^=0.507, adjusted *R*^2^=0.395, F_5,22_=7.714; *P*<.001.

^c^Not applicable.

^d^Indicates significant values.

## Discussion

### Principal Findings

In this study, we quantified the relationship between the proportion of fake news, its propagation, and vaccination decisions in Taiwan, using multivariable linear regression and interaction analysis. A higher percentage of fake news about COVID-19 and vaccines on the internet and greater search volumes predicted more adverse effects on vaccination doses administered in the following week. During the study interval, the fake news percentage threshold was 37.4%, which was the zero-crossing coefficient of the Google Trends level and was statistically significant when it reached 39.3%. This number may vary with study intervals, but this trend existed even in the unseen validation data. The exposure of populations to more than a specific amount of fake news about diseases and vaccines can negatively impact public health. Public health work on vaccination should strengthen public immunity to fake news and encourage balance and objectivity among news media outlets.

The overall percentage of fake news rose by 2 points during the public vaccination stage. One reason for this increase might be the official announcement of the community spread of COVID-19 in Taiwan on May 15, 2021, although there was no specifically significant increase in the fake news percentage for the following 2 weeks (26,447/73,669, 35.9%; 95% CI 35.6%-36.3%). The percentage increased significantly during the first 10 days of June 2021 (19,969/52,276, 38.2%; 95% CI 37.8%-38.6%). At the same time, Taiwan was facing its second peak of infection and received Japan's donation of the first batch of vaccines. The number of infections then ebbed, but some media outlets seemed to still overreact during the public vaccination stage (Multimedia Appendix 3). News media have different news styles based on their culture, which might relate to varying levels of suspicion and incitement. Figure 5 shows the different fake news percentages for each form of media, some of which maintained a consistent style in both stages, but some of which increased significantly in the second stage. The greatest increase was 1.7 times and the second largest was a 34% increase. Lazer et al [13] indicated that the internet accelerated the news media's move toward biased and affective reporting. Internet news outlets are commercial, and click-through rates reflect revenue and sometimes share prices. Using attractive discourse and sentimental titles will be the preference of some media companies, and sometimes the content is subjective and lacks fact-checking. It may be reasonable to change styles in the pursuit of click-through rates, but this approach might undermine the credibility of the media and public trust.

The number of vaccine doses available had a positive adjusted effect on the number of vaccine doses administered in the following week. For most people seeking vaccinations in Taiwan, it is necessary to reserve a vaccination day and then visit. As when booking a flight, the number of seats on an aircraft determines the number of bookings available. Although no-shows happen, overselling is prohibited when it comes to vaccination, as limited resources could lead to a “bank run” phenomenon on vaccination, especially if the masses panic. In August 2021, fewer than 400,000 vaccine doses were available every week, and the rate of vaccination was slow without the “vaccine run” effect (Figure 1). In that month, the percentage of fake news increased 1 point to 38.5% (95% CI 38.2%-38.8%), exceeding the threshold, but not reaching a significant level, which may be a decelerating factor.

In the regression model analysis, we factored out infection and death cases, because COVID-19 was gradually brought under control over the interval analyzed, and the number of deaths was correlated with the number of infections. We found multicollinearity between the number of infections and the percentage of fake news, the Google Trends level, and their interaction term. The values for infection cases could be almost linear combinations of these factors, potentially undermining the reliability of the model. The coefficients for these factors were 1.0, –0.7, and 2.9 respectively (*R*^2^=0.896; *P*<.001).

In this study, we used the Google Trends level to represent the magnitude of the spread of COVID-19 and vaccine news. We believe this approach is justifiable because Google’s dominance of the market share of searches makes them a good proxy for the overall data. The trend for declining search levels within the study interval may be related to the ebb of COVID-19 infections and the public's attention shifting to other issues. These tendencies might reflect a link between information dissemination and the Google Trends level. It is a caveat to note that the Google Trends tool does not provide consistent results; specifically, the Google Trends level varies based on the selected time interval and is relative over time rather than being a fixed score. In the regression analysis, normalization was used to counteract this variation in the data. The effects of the subgroups of COVID-19 and vaccine news on vaccination were also analyzed, but only the entire set had statistically significant results. When people search for information about vaccines, relevant information will be available to the public through associated links, search engines, or recommender systems. Everyone is faced with an overwhelming amount of information on the internet, few people read every news item, and sometimes people skim them. Also, attention may shift to another related topic rather than the original one during a search [49]. These sources of noise might lead to a lack of statistical significance when using news subgroups with only COVID-19 or vaccines.

The interaction between the fake news percentage and the Google Trends level is an important factor in this regression analysis, without which no statistical significance can be observed for the individual variables. This observation may suggest that no matter what the media has to offer, it cannot influence public opinion without human contact. However, this lack of access is not possible unless the internet collapses. In this study, we found that there is a threshold above which the fake news percentage had a negative impact, which might be regarded as the point at which the resistance of the public to misinformation was overcome. As more media outlets adopt attractive journalism styles and more inciting discourse, it may be practical to strengthen our resistance rather than restrict the freedom of expression of the media, but the media should reflect and consider returning to the essence of journalism.

### Comparison With Prior Works

Lazer et al [13] points out that little is known about the prevalence of misinformation or the scale of its spread and impact. To the best of our knowledge, studies to date have not explicitly addressed these gaps. Loomba et al [22] designed a prospective study to examine vaccine intent before and after exposure to misinformation and confirmed that misinformation has adverse effects on vaccination rates. Questionnaire studies have demonstrated the impact of misinformation on vaccine hesitancy [20-22], but this approach does not quantify how much misinformation is needed to change the public’s perspective. King and Wang [24] retrospectively collected 42 million tweets and found that messages containing misinformation or emotional content spread quickly. Infodemic research involving social media data is common [23,24], and information about user interactions can be used to analyze the dissemination of information. The amount of misinformation can be estimated from public postings, but this approach may lead to an underestimation of the extent of the misinformation because the data do not include information from private communities or groups on social media.

This study used big news data, and the target population was the population of Taiwan. The results were consistent with those from previous studies [20-22], which found that misinformation can lower vaccine intent. We further quantified the effect of varying amounts of fake news on the public vaccination rate. By accessing almost every news outlet in Taiwan, we estimated the prevalence of fake news using an automatic style-based detection method. Although we adopted a broad definition of fake news, the results of this study provided an estimate of the extent of fake news in Taiwan. However, the best way to directly estimate the spread of misinformation remains a challenge.

### Implications

The internet connects the world, shortening the distance between people by the rapid transfer of information. Computers have shrunk to the size of a palm, and in the information society, most people can surf the internet anytime and anywhere. During the last few decades, many economic activities and startups have flourished with the benefit of the internet. These organizations provide as much information as we can imagine for free or very cheaply. Much knowledge and information are open source and can enhance our abilities or interfere with our decision-making based on the way we use it. As more and more well-designed open-source generative language models become available, large amounts of unverified information may shortly be packaged by bots as attractive news on the web. Sometimes bots are designed for a specific issue [13] and might have malicious intent. The growth of biased, intentional, or extremist public opinion in the news is sometimes difficult to detect, but it potentially impacts our thinking [25,26]. Understanding the potential media framing is a vital personal ability in the internet age of massive information floods.

Some online resources are available for fact-checking [26], providing the public with access to media literacy. While the Islander system cannot directly detect false information, it can monitor the media in real time and provide objective scores. These scores help us think critically; identify the opinions, roles, and goals of the media; and determine whether an item of information is credible. The news analysis systems work like an attenuated vaccine, reducing the toxicity of malicious information, increasing our immunity to misinformation, and preventing the spread of fake news. Future work on this issue should focus on providing a progressively more robust information judgment system that can grow with fake news generators even under adversarial attacks.

### Limitations

One limitation of this study is the lack of detailed demographic information about vaccination recipients, as a result of which we could not investigate further factors that influence vaccination decisions. The scope of the study was to investigate the relationship between digital news and vaccination decisions, and some demographic characteristics that may be relevant for accessing web news. The lack of such detailed information makes it challenging to explore consumer engagement with digital media. Another limitation is that this study was conducted in an Asian society, and the news judgment system is only applicable to Chinese news, which makes it difficult to adapt the results and web applications to another region or society. Nevertheless, in recent years, dubiousness in digital news has become an important global issue, and the results of this study revealed its implications for vaccination in Asian societies. In future works, such news analysis systems may be established in different regions to help enhance the media literacy of the public, while collecting news data in different areas and conducting extended analyses.

### Conclusions

In this study, we retrospectively analyzed an Asian society of 23 million people, using deep learning NLP methods to analyze 0.7 million digital news items over a half-year period, and identified a correlation between the percentage of fake digital news and COVID-19 vaccination doses. A higher prevalence of fake news had a significantly more adverse effect on vaccination decisions. Public health policy efforts to increase vaccination coverage might focus on reducing the impact of fake news on the public, and the use of news analysis systems may help to improve the public's media literacy.

## Appendix

Multimedia Appendix 1 Meaning and English translation of search keywords, and information on digital media sources.

Multimedia Appendix 2 Distribution of scores. The left side is the suspicion score distribution; the dotted line indicates a Poisson distribution. On the right is the incitement score distribution; the dotted line represents a Gaussian distribution.

Multimedia Appendix 3 Percentage trend of suspicious news in some media sources.

## Abbreviations

CVAT: Chinese valence-arousal text data set
NLP: natural language processing
