# Supplementary material for: The Prevalence and Impact of Fake News on COVID-19 Vaccination in Taiwan: Retrospective Study of Digital Media
Source: J Med Internet Res. 2022 Apr 26;24(4):e36830. doi: 10.2196/36830 (PMC9045486; doi:10.2196/36830)
Supplement: Multimedia Appendix 1 [file jmir_v24i4e36830_app1.pdf]

Appendices

Appendix1- Table 1. Meaning and English translation of search keywords.

**Keyword**	**English translation**	**Meaning as a keyword for news searches**
破口	Break	Infection control breach.
病例	Confirmed case	The COVID-19 cases.
放寬	Relax	Ease the lockdown restrictions.
疫	Epidemic	COVID-19 pandemic.
隔離	Isolation/ quarantine	Measures to contain the coronavirus.
確診	Confirmed case	The COVID-19 cases.
新冠	COVID-19	Coronavirus disease 2019
新型冠狀病毒	COVID-19	Coronavirus disease 2019
肺炎	Pneumonia	Lung infection with inflammatory changes
疾管	Disease Control	Chinese abbreviation for centers for disease control (CDC)
疫苗	vaccine	Used to provide immunity against COVID-19.
高端	Medigen MVC Vaccine	One COVID-19 vaccine brand name.
默德納	Moderna	One COVID-19 vaccine brand name.
接種	Vaccination	Vaccine uptake.

Appendix1- Table 2. Digital media information. (Descending alphabetically. The order of news sources does not correspond to Figure 5.)

Digital media	URL
紐約時報中文網（The New York Times Chinese - Traditional)	https://cn.nytimes.com/
工商時報（Commercial Times)	https://ctee.com.tw/
中天電視（CTI Television)	https://gotv.ctitv.com.tw/
經濟日報（Economic Daily News)	https://money.udn.com/
公視新聞網（Public Television)	https://news.pts.org.tw/
TVBS	https://news.tvbs.com.tw
新頭殼（Newtalk)	https://newtalk.tw/
蘋果日報（Apple Daily)	https://tw.appledaily.com
Yahoo 奇摩（Yahoo News)	https://tw.news.yahoo.com/
聯合新聞網（United Daily News)	https://udn.com/news/
中時電子報（China Times)	https://www.chinatimes.com
信傳媒（CredereMedia)	https://www.cmmedia.com.tw/
中央社（Central News Agency)	https://www.cna.com.tw/
華視新聞網（Chinese Television System)	https://www.cts.com.tw/
CTWANT	https://www.ctwant.com/
大紀元（The Epoch Times)	https://www.epochtimes.com/
年代新聞網（Era News)	https://www.eracom.com.tw/EraNews/
ETtoday 新聞雲（ETtoday News)	https://www.ettoday.net/
民視新聞（Formosa News)	https://www.ftvnews.com.tw/
自由時報（The Liberty Times)	https://www.ltn.com.tw/
鏡週刊（Mirror Media)	https://www.mirrormedia.mg/
今日新聞（NOW News)	https://www.nownews.com/
民報（Taiwan People News)	https://www.peoplemedia.tw/
SETN (Sanlih E-Television)	https://www.setn.com/
風傳媒（The Storm Media)	https://www.storm.mg/
上報（UP Media)	https://www.upmedia.mg
