# Supplementary figures and images for: The Prevalence and Impact of Fake News on COVID-19 Vaccination in Taiwan: Retrospective Study of Digital Media
Source: J Med Internet Res. 2022 Apr 26;24(4):e36830. doi: 10.2196/36830 (PMC9045486; doi:10.2196/36830)

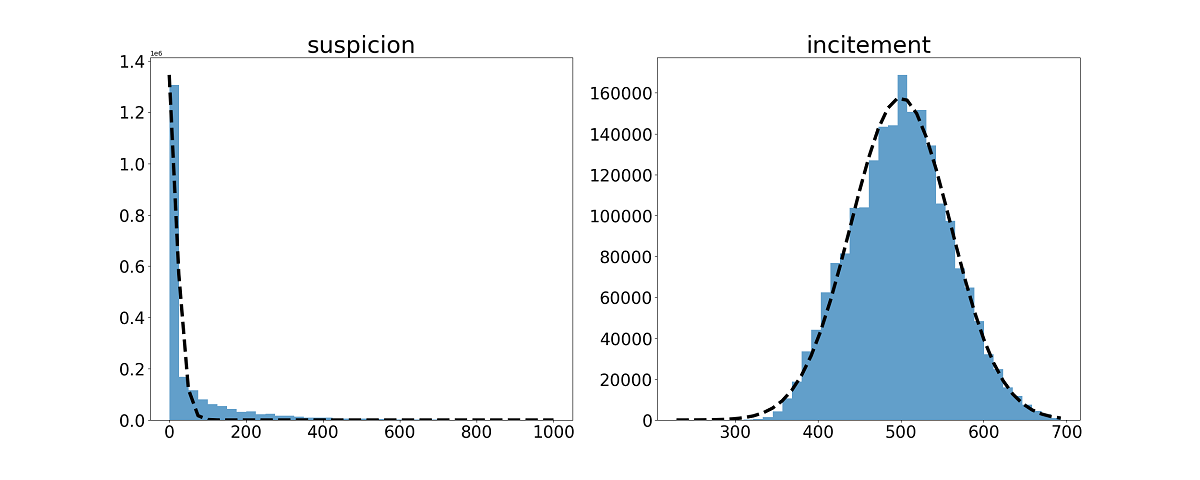

Supplement: Multimedia Appendix 2 [file jmir_v24i4e36830_app2.png]

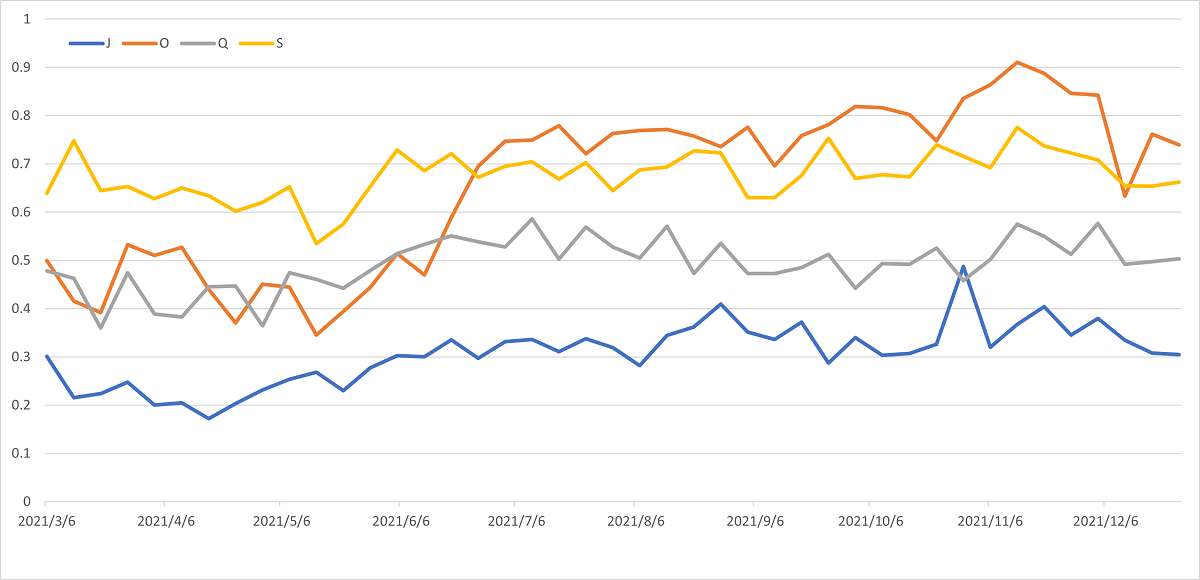

Supplement: Multimedia Appendix 3 [file jmir_v24i4e36830_app3.png]
